# Association between vitamin D and zoledronate-induced acute-phase response fever risk in osteoporotic patients

**DOI:** 10.3389/fendo.2022.991913

**Published:** 2022-10-10

**Authors:** Ke Lu, Qin Shi, Ya-qin Gong, Chong Li

**Affiliations:** ^1^ Department of Orthopedics, Affiliated Kunshan Hospital of Jiangsu University, Suzhou, China; ^2^ Department of Orthopedics, Gusu School, Nanjing Medical University, Suzhou, China; ^3^ Department of Orthopedics, the First Affiliated Hospital of Soochow University, Orthopedic Institute of Soochow University, Suzhou, China; ^4^ Information Department, Affiliated Kunshan Hospital of Jiangsu University, Suzhou, China

**Keywords:** vitamin D, zoledronate, acute-phase response (APR), osteoporosis, fever

## Abstract

**Objectives:**

To elucidate the independent correlation between vitamin D content and zoledronate (ZOL)-triggered acute-phase response (APR) fever risk in osteoporotic (OP) patients, and to examine the potential threshold for optimal vitamin D concentrations that prevent the occurrence of ZOL-induced fever.

**Methods:**

This retrospective investigation was based on a prospectively documented database compiled at the Affiliated Kunshan Hospital of Jiangsu University between January 2015 and March 2022. In total, 2095 OP patients, who received ZOL during hospitalization, were selected for analysis. The primary endpoint was the presence (>37.3°C) or absence (≤37.3°C) of fever, quantified by the maximum body temperature, measured within 3 days of ZOL infusion. The exposure variable was the baseline serum 25-hydroxyvitamin D (25[OH]D) levels.

**Results:**

The OP patients with fever exhibited markedly reduced 25(OH)D content than those without fever. Upon adjusting for age, gender, order of infusion of ZOL, main diagnosis, season of blood collection, year of blood collection, calcitonin usage, and beta-C-terminal telopeptide of type I collagen (β-CTX) levels, a 10 ng/mL rise in serum 25(OH)D content was correlated with a 14% (OR, 0.86; 95% CI, 0.76 to 0.98, *P*-value = 0.0188) decrease in the odds of ZOL-induced fever. In addition, a non-linear relationship was also observed between 25(OH)D levels and fever risk, and the turning point of the adjusted smoothed curve was 35 ng/mL of serum 25(OH)D content.

**Conclusions:**

Herein, we demonstrated the independent negative relationship between serum 25(OH)D content and ZOL-induced fever risk. According to our analysis, 25(OH)D above 35 ng/mL may be more effective in preventing ZOL-induced APR. If this is confirmed, a “vitamin D supplemental period” is warranted prior to ZOL infusion, particularly the first ZOL infusion, to ensure appropriate 25(OH)D levels that protect against ZOL-induced fever.

## Introduction

Osteoporosis (OP) is a chronic, progressive disease that manifests as low bone mass, degenerated bone micro-architecture, as well as enhanced bone fragility and fracture risk (1). Based on a 2018 Chinese epidemiological survey, 19.2% of OP patients were above 50 years old, and 32.0% of the Chinese population above 65 years old was diagnosed with OP (2). Among the numerous fragility fractures associated with OP, vertebral and hip fractures are the most correlated with enhanced morbidity and mortality (3). Based on the current statistics, around 30–50% females and 15–30% males will likely experience OP-related fracture in their lifetime (4, 5). Therefore, early detection of high-risk patients, and subsequent treatment, is most crucial to the health and quality of life of these patients.

Bisphosphonates (BPs) are widely used for treating OP, and they are reported to markedly reduce osteoclast-based bone resorption, thus diminishing the potential for vertebral and non-vertebral fractures (6). Zoledronate (ZOL) 5 mg is an intravenous BP, administered once a year, and it was approved for treating and preventing postmenopausal or glucocorticoid-induced OP, as well as enhance bone mass in OP males. Since it is infused once a year into patients, the patient adherence to ZOL is exceptionally high, and it avoids gastrointestinal absorption/irritation challenges commonly observed with oral BPs (7). Despite this, there are reports of transient acute phase reactions (APRs) like fever, myalgia, and flu-like symptoms during the first 3 days post first ZOL administration (8). To ensure enhanced patient adherence, it is critical to develop approaches that either prevent or better manage these APRs in patients receiving their first ZOL infusion.

Prior investigations identified certain APR risk factors, which can serve as indicators for APR development (9, 10). Among these factors, appropriate serum 25-hydroxy vitamin D (25[OH]D) content prior to ZOL infusion was demonstrated to markedly reduce APRs incidence (11–13). However, apart from clinical trials, the real world medical evidences on the independent correlation between serum 25(OH)D levels and APR risk in large patient populations are relatively scarce. In addition, there is no standard cut-off for appropriate 25(OH)D concentrations and ZOL-induced APR risk prior to ZOL infusion. Thus, the goal of our research was to further explore the correlation between serum 25(OH)D content and APR risk in the clinic.

## Materials and methods

### Study design and subject selection

This retrospective investigation utilized a prospectively collected database (January 2015-March 2022) at the Affiliated Kunshan Hospital of Jiangsu University located in Kunshan in eastern China (31.2°N latitude, around 30 kilometers from Shanghai). OPFs, also known as fragility fractures, are low-energy fractures that result from a fall from a standing height or less, and they can markedly enhance the risk of future fragility fractures (14). OP diagnosis is based upon the presence of OPFs, and simultaneous absence of additional metabolic bone disorders. OP can even occur in patients with normal bone mineral density (T-score). Alternately, OP diagnosis can also be dependent on a T-score of −2.5 or less, in a patient who has not suffered any relevant fractures (15). Owing to the medical insurance payment, a vast majority of OP patients require hospitalization to receive ZOL in the Kunshan City. According to the management protocol of ZOL infusion in our hospital and due to the renal function-protective effect of hydration (16), patients with normal cardiac activity were typically administered with 500ml normal saline before and after ZOL administration The duration of ZOL administration (5 mg, 100mL) was more than 30 minutes, except for the duration administration of normal saline. Patients with poor heart condition received half or less of the amount of normal saline provided to patients with normal heart activity. In terms of OP patients with new fragility fractures, APR interference (judged by postoperative fever) following ZOL infusion was avoided by only administering ZOL prior to discharge, once the postoperative condition stabilized. This typically took 7-14 days post orthopedic surgery. If postoperative infection was suspected, ZOL administration was delayed. These patients were then contacted, and reminded by our fracture liaison service (FLS) team, of their ZOL infusion at the 3-month follow up.

**Figure 1:**
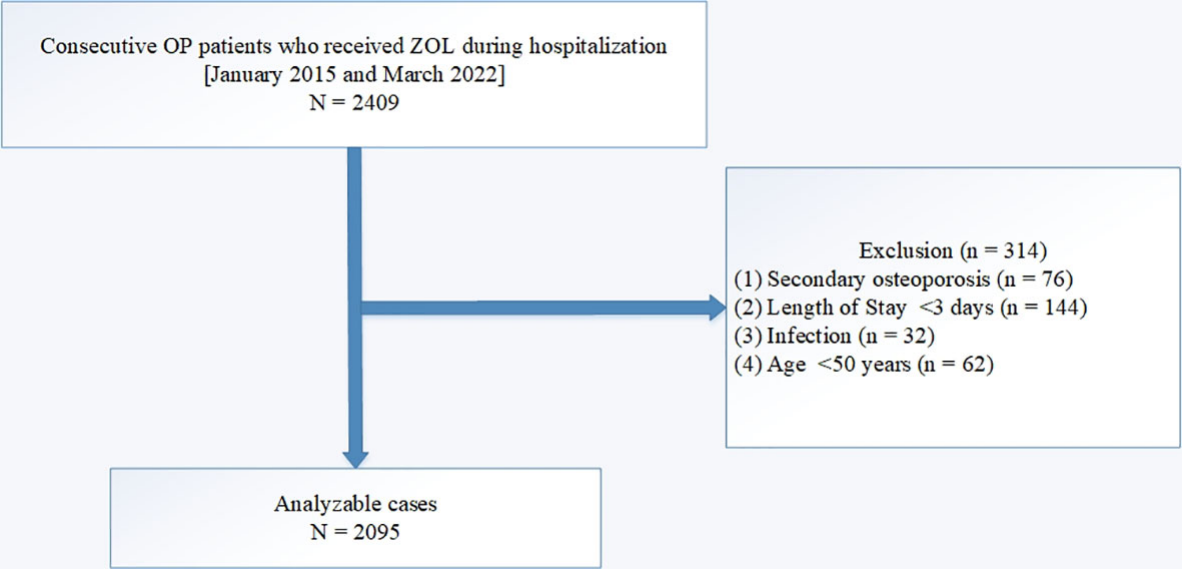
A schematic of our study design.

Consecutive 2409 OP patients, who received ZOL during hospitalization, were recruited for analysis. The following patients were excluded from analysis: 1) secondary OP (n = 76); b) length of stay <3 days (n = 144); c) infection (n = 32), and e) age <50 years (n = 62). Overall, 2095 patients were finally selected for analysis, according to our inclusion and exclusion criteria. A schematic diagram of our patient selection process is presented in **Figure 1**. We received ethical approval from the Affiliated Kunshan Hospital of Jiangsu University (approval No. 2020-03-046-K01), and the study strictly followed the Declaration of Helsinki. The analyzed patient data was initially recorded as part of the hospital’s quality improvement services. Researchers who analyzed the data were blinded to the patient identification information. The requirement for signed informed consent was waived due to the anonymous and observational design of this investigation.

### Outcome

Fever is one of the five symptoms of APR (8). Its probability is the highest among all APR symptoms, and it can be quantitatively and objectively monitored. Hence, we selected fever as our dependent variable and the primary outcome. The axillary body temperature of each patient was routinely recorded at 6:00 and 14:00 on days 1 to 3 post ZOL infusion. If a patient’s axillary temperature was above 37.3°C, then the measurement was repeated every four hours. Finally, the maximum temperature, measured within the 3 days post ZOL infusion, was included in the analysis. Based on the threshold provided by prior investigations (10, 17), we altered the continuous temperature variable to a dichotomous variable in our models. Hence, in this study, ≤37.3°C represented no fever, and >37.3°C represented fever.

### Exposure variable

Herein, the exposure variable was vitamin D. In humans, the most common form of vitamin D is serum 25(OH)D, and prior reports suggested that serum 25(OH)D content can accurately predict a patient’s vitamin D status. The baseline serum 25(OH)D content was recorded immediately upon admission using an automated electrochemiluminescence immunoassay on a Roche Cobas 8000/e602 analyzer (Roche Diagnostics, Mannheim, Germany). Currently, there is no standard 25(OH)D threshold value for determining APR risk. However, the Institute of Medicine (National Academy of Sciences, Washington, DC, USA) and the National Osteoporosis Society (Bath, England) agreed that a serum 25(OH)D content of (2.5 nmol/L 25[OH]D = 1 ng/mL 25[OH]D) of < 30 nmol/L (12ng/mL) is deficient, 30-50 nmol/l (12-20 ng/mL) is insufficient for certain individuals, and >50 nmol/L (20 ng/mL) is sufficient for most individuals (18, 19). The time of blood collection was also analyzed in this study. Lastly, seasons were described as: Spring, March–May; Summer, June–August; Autumn, September–November; and Winter, December–February.

### Covariates

The following covariates were also considered in our analysis: age, gender, body mass index (BMI), order of ZOL infusion, season of blood collection, year of blood collection, main diagnosis (OP without fractures/OPF), hypertension, diabetes, comorbidity, calcitonin usage, alendronate (ALEN) usage, nonsteroidal anti-inflammatory drug (NSAID) usage, statins usage, hemoglobin, neutrophil count, peripheral blood lymphocyte count, monocyte count, platelet count, serum total calcium, alanine transaminase, creatinine, beta-C-terminal telopeptide of type I collagen (β-CTX), procollagen type I N-terminal propeptide (P1NP), ferritin, total cholesterol, triglyceride, high-density lipoprotein, low-density lipoprotein, and homocysteine. All laboratory variables were measured within 3 days prior to ZOL treatment, and patients were fasted for 8 hours prior to blood sample collection. Calcitonin, ALEN, NSAID, and statins usages were defined as administration prior to ZOL infusion. Calcitonin was provided daily 50 IU *via* subcutaneous or intramuscular injection. Comorbidity was assessed using the Charlson comorbidity index (CCI) (20).

### Statistics

Patient demographics, clinical, and laboratory characteristics are presented as mean (standard deviation [SD]) or/and median (first quartile [Q1] to third quartile [Q3]) in terms of continuous data, and as frequency (percentage) in terms of categorical data. A univariate analysis of categorical data was carried out *via* the Pearson’s chi-square or Fisher’s exact test. Normally distributed continuous data were assessed *via* the independent samples t-test, and non-normally distributed data *via* the Mann-Whitney U test. Furthermore, univariate logistic regression was conducted to screen potential relationships between the OP patient profiles and ZOL fever risk. Using generalized estimating equations (GEE), we further assessed the independent relationship between ZOL-induced fever risk and serum 25(OH)D in OP patients, while controlling for covariance influences. We compared the outcomes of the unadjusted (crude model) or minimally adjusted model (Model I) with those from fully-adjusted model (Model II/III). Firstly, we conducted collinearity covariance diagnoses *via* variance inflation factor (VIF) analysis. Next, we assessed the need for covariance adjustment based on the following: Criteria 1, a covariate was introduced to the basic model or removed from the full model, and the matched odds ratio (OR) was altered by a minimum of 10%; Criteria 2: Criteria 1 or a covariate *P*-value of <0.1, based on the univariate model (21). Hence, in case of fully-adjusted models, Model II was established according to Criteria 1, and Model III utilized Criteria 2.

We further established non-linear relationships using a generalized additive model (GAM). Once a non-linear relationship was identified, the cut-off (to smooth the curve) was computed *via* a two-piecewise linear regression model. Upon the detection of a clear ratio in the smoothing curve, the recursive technique was employed to automatically compute the turning point for the maximum likelihood model (22).

Furthermore, to examine subgroup robustness and potential variation, we repeated the subgroup analyses while classifying various covariates. The subgroup modifications and associations were further compared using the likelihood ratio test (LRT).

We also conducted a sensitivity analysis to determine the impact of the APR evaluation approach. APR evaluation was done *via* C-reactive protein (CRP) measurement within 3 days post ZOL administration, and not body temperature measurement. The continuous CRP data was then classified as the dichotomous data as follows: ≤0.8 mg/dL represented CRP^-^, and >0.8mg/dL represented CRP^+^.

The Empower Stats (www.empowerstats.com, X&Y solutions, Inc., Boston, MA, USA) and R software version 3.6.3 (http://www.r-project.org) were employed for all data analyses. A *P*-value<0.05 was deemed as significant.

## Results

### Patient profile and univariate analysis for fever risk

**Table 1** lists our patient characteristics. The average patient age was 68.43 years old, and 83.87% (n = 1757) were female. The maximum axillary body temperature measured within the first three days following ZOL infusion at the threshold of >37.3°C was detected in 39.11% of patients. The fever incidences were 45.73%, 13.65%, 5.26%, and 11.11% following the 1st, 2nd, 3rd, and 4th ZOL infusions, respectively (*P*-value for trend<0.001). The average (SD) serum 25(OH)D content was 21.15 (8.84) ng/mL. Moreover, serum 25(OH)D concentration exhibited seasonal variation, with peak in September and trough in March. The combined season of summer and autumn (June to November) revealed a markedly higher serum 25(OH)D concentration (β, 2.69; 95% confidence interval [CI], 1.94 to 3.44; *P*-value<0.0001), compared with the combined season of winter and spring (December to May) (**Table S1**). The mean (95% CI) serum 25(OH)D levels were 20.47 (20.05 to 20.89) ng/mL, 23.47 (22.52 to 24.42) ng/mL, 25.13 (23.21 to 27.06) ng/mL, and 29.40 (23.69 to 35.10) ng/mL prior to the 1^st^, 2^nd^, 3^rd^, and 4^th^ ZOL infusions, respectively (*P*-value for trend<0.001).

**Table 1:** Characteristics of study participants and univariate analysis for ZOL-induced fever risk.

Variables	(N) Mean (SD) Median (Q1-Q3)^a^	OR^b^ (95% CI) *P*-value
Temperature continuous^c^, °C	(2038) 37.28 (0.68) 37.20 (36.80-37.50)	
Fever, N (%)		
No (Temperature <=37.3°C)	1241 (60.89%)	
Yes (Temperature >37.3°C)	797 (39.11%)	
25(OH)D level continuous, ng/mL	(2095) 21.15 (8.84) 20.00 (15.00-25.89)	0.97 (0.96, 0.98) <0.0001
25(OH)D level categorical, N (%)		
Deficiency (<12 ng/mL)	235 (11.22%)	Reference
Inadequacy (>=12, <20 ng/mL)	788 (37.61%)	0.99 (0.74, 1.33) 0.9487
Adequacy (>=20 ng/mL)	1072 (51.17%)	0.63 (0.47, 0.84) 0.0016
Order of ZOL infusion, N (%)		
1st	1681 (80.24%)	Reference
2nd	326 (15.56%)	0.19 (0.13, 0.26) <0.0001
3rd	79 (3.77%)	0.07 (0.02, 0.18) <0.0001
4th	9 (0.43%)	0.15 (0.02, 1.19) 0.0723
Season of blood collection, N (%)		
Spring (March, April and May)	519 (24.77%)	Reference
Summer (June, July and August)	518 (24.73%)	0.98 (0.76, 1.27) 0.8880
Autumn (September, October and November)	612 (29.21%)	0.89 (0.70, 1.14) 0.3559
Winter (December, January and February)	446 (21.29%)	1.11 (0.85, 1.44) 0.4540
Year of blood collection, N (%)		
2015	21 (1.00%)	Reference
2016	32 (1.53%)	3.60 (0.87, 14.84) 0.0763
2017	48 (2.29%)	5.52 (1.44, 21.23) 0.0129
2018	129 (6.16%)	5.91 (1.66, 21.04) 0.0061
2019	483 (23.06%)	5.13 (1.49, 17.66) 0.0095
2020	583 (27.83%)	4.03 (1.18, 13.85) 0.0267
2021	706 (33.69%)	2.73 (0.80, 9.36) 0.1105
2022	93 (4.44%)	4.73 (1.30, 17.17) 0.0181
Gender, N (%)		
Male	338 (16.13%)	Reference
Female	1757 (83.87%)	0.77 (0.61, 0.98) 0.0310
Age, y	(2095) 68.43 (8.73) 68 (63-74)	1.00 (0.99, 1.01) 0.6552
BMI, kg/m^2^	(2095) 23.43 (3.11) 23.55 (21.48-24.97)	
Main diagnosis, N (%)		
OP without fractures	1422 (67.88%)	Reference
OPF	673 (32.12%)	3.96 (3.26, 4.81) <0.0001
Hypertension, N (%)		
No	1438 (68.64%)	Reference
Yes	657 (31.36%)	0.77 (0.63, 0.93) 0.0073
Diabetes, N (%)		
No	1799 (85.87%)	Reference
Yes	296 (14.13%)	0.83 (0.64, 1.07) 0.1430
CCI^d^ score	(2095) 0.98 (2.17) 0 (0-1)	0.96 (0.92, 1.01) 0.0921
Calcitonin usage, N (%)		
No	1685 (80.43%)	Reference
Yes	410 (19.57%)	3.33 (2.65, 4.17) <0.0001
Alendronate usage, N (%)		
No	2083 (99.43%)	Reference
Yes	12 (0.57%)	0.52 (0.14, 1.92) 0.3238
NSAIDs usage, N (%)		
No	2054 (98.04%)	Reference
Yes	41 (1.96%)	1.50 (0.81, 2.78) 0.2026
Statins usage, N (%)		
No	2074 (99.00%)	Reference
Yes	21 (1.00%)	0.78 (0.31, 1.93) 0.5867
Hemoglobin, g/L	(2025) 123.86 (16.32) 125.80 (116-134)	0.98 (0.98, 0.99) <0.0001
Neutrophil count, ×10^9^/L	(2024) 4.32 (2.36) 3.70 (2.70-5.02)	1.22 (1.17, 1.28) <0.0001
Lymphocyte count, ×10^9^/L	(2024) 1.43 (0.56) 1.40 (1.04-1.77)	0.58 (0.49, 0.69) <0.0001
Monocyte count, ×10^9^/L	(2024) 0.41 (0.19) 0.40 (0.30-0.50)	3.74 (2.31, 6.05) <0.0001
Platelet count, ×10^9^/L	(2024) 184.76 (61.92) 179.00 (144-217)	1.00 (1.00, 1.00) 0.8358
Calcium, mmol/L	(2059) 2.26 (0.17) 2.26 (2.17-2.35)	0.11 (0.06, 0.20) <0.0001
Alanine transaminase, U/L	(2072) 21.24 (15.51) 17 (13-25)	1.01 (1.00, 1.01) 0.0238
Creatinine, μmol/L	(2071) 61.20 (24.19) 56 (49-67)	1.00 (0.99, 1.00) 0.0549
β-CTX, ng/mL	(2075) 0.42 (0.30) 0.33 (0.19-0.58)	5.28 (3.83, 7.29) <0.0001
P1NP, ng/mL	(2074) 50.27 (34.52) 43 (29-63)	1.01 (1.01, 1.01) <0.0001
Ferritin, ng/mL	(1683) 258.7 (266.7) 196.8 (125.4-310.5)	1.001 (1.000, 1.001) 0.0002
Total cholesterol, mmol/L	(1595) 4.6 (1.0) 4.5 (3.9-5.2)	1.00 (0.89, 1.12) 0.9606
Triglyceride, mmol/L	(1595) 1.4 (0.9) 1.1 (0.8-1.6)	0.91 (0.82, 1.01) 0.0633
High-density lipoprotein, mmol/L	(1595) 1.5 (0.3) 1.4 (1.2-1.7)	0.70 (0.52, 0.96) 0.0249
Low-density lipoprotein, mmol/L	(1595) 2.7 (0.8) 2.7 (2.2-3.2)	0.91 (0.80, 1.03) 0.1409
Homocysteine, μmol/L	(1596) 13.0 (6.4) 11.4 (9.3-15.1)	1.00 (0.99, 1.02) 0.6305

a Continuous variables.

b Dependent variable fever, as a result of univariate analysis for ZOL-induced fever risk.

c Maximum axillary body temperature, measured within the first three days after ZOL infusion.

d Higher scores indicate more severe comorbidities.

SD, standard deviation; Q1, first quartile; Q3, third quartile; OR, odds ratio; CI, confidence interval; BMI, body mass index; β-CTX, beta-C-terminal telopeptide of type I collagen; P1NP, procollagen type I N-terminal propeptide; 25(OH)D, 25-hydroxy vitamin D; NSAID, nonsteroidal anti-inflammatory drug; OP, osteoporosis; OPF, osteoporotic fracture; CCI, Charlson comorbidity index; ZOL, zoledronate.

**Figure 2:**
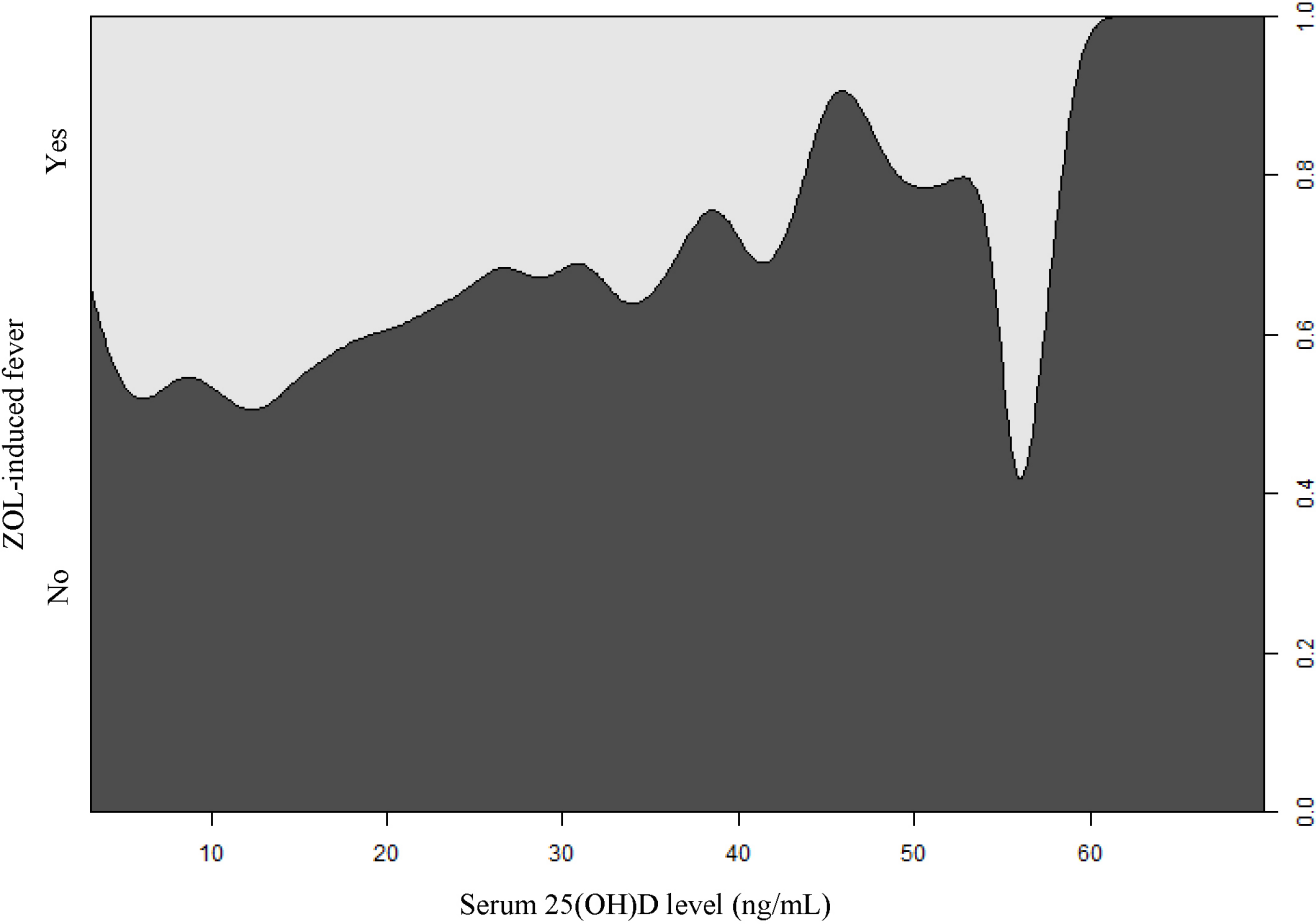
The conditional density plot depicting the correlation between serum 25(OH)D levels and ZOL-induced fever. 25(OH)D, 25-hydroxy vitamin D; ZOL, zoledronate.

**Figure 3:**
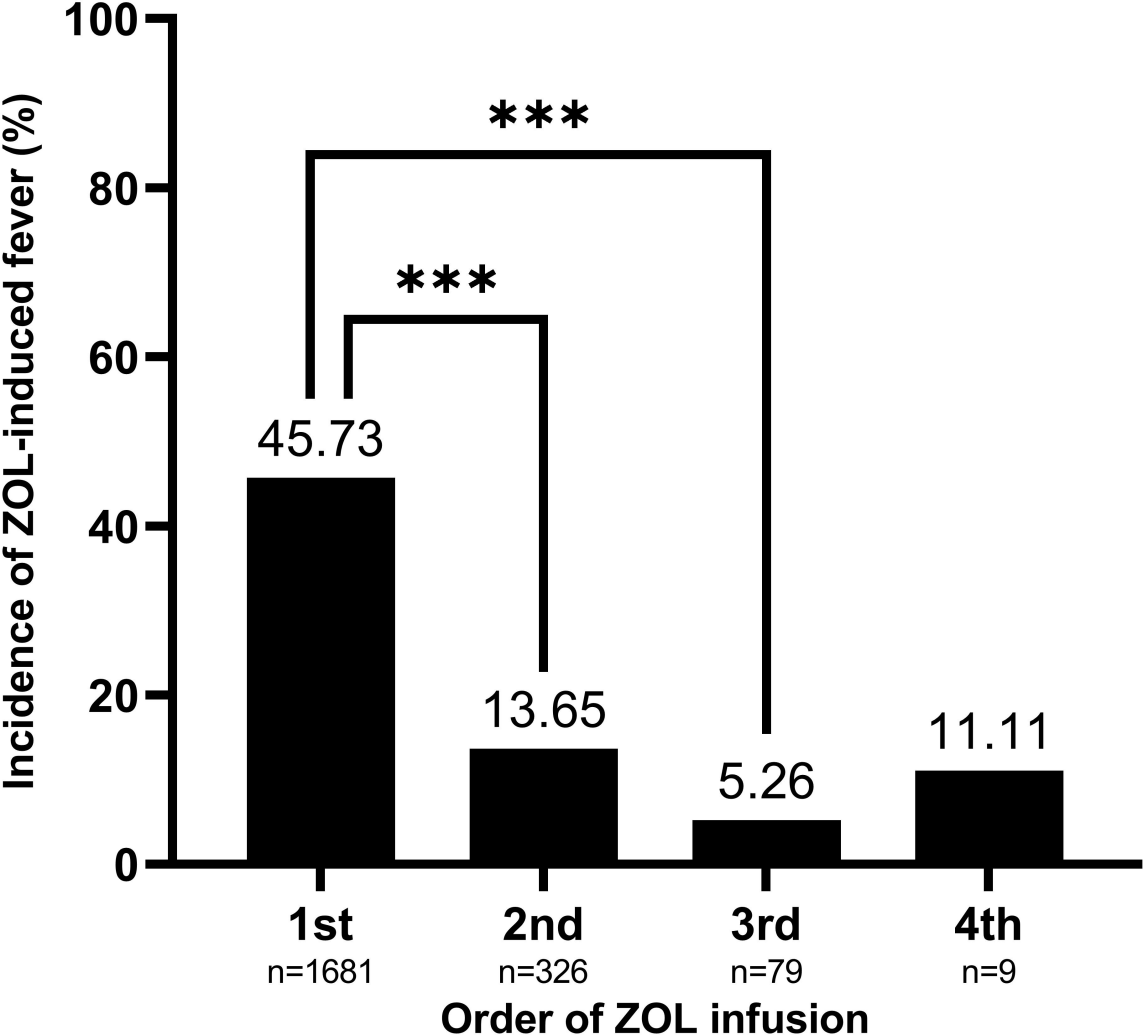
The order of ZOL infusion depicting a significantly negative association with ZOL-induced fever risk. Data are presented as mean. ***P-value<0.0001 as indicated. ZOL, zoledronate.

Based on our univariate analysis, serum 25(OH)D levels were negatively correlated with fever risk (OR, 0.97; 95% CI, 0.96 to 0.98; *P*-value <0.0001), as shown in **Table 1** and **Figure 2**. We also explored the relationships between covariates and fever risk in our univariate analysis (**Table 1**). Firstly, the ZOL infusion order revealed a significantly negative association with fever risk. **Figure 3** illustrates the incidence of ZOL-induced fever following the first and subsequent ZOL infusions. Secondly, women exhibited a 23% lower ZOL fever risk, compared to men (*P*-value = 0.0073). Thirdly, a significantly reduced incidence of fever was seen in October (OR, 0.53; 95% CI, 0.34 to 0.84; *P*-value = 0.0073). Fever risk was also significantly reduced in November (OR, 0.62; 95% CI, 0.40 to 0.97; *P*-value = 0.0342) (**Table S2**). Fourthly, relative to OP patients without fractures, OPF patients exhibited a higher fever risk (OR, 3.96; 95% CI, 3.26 to 4.81; *P*-value<0.0001). Furthermore, upon categorizing OPF by fracture site, vertebral fractures exhibited the highest fever risk, compared to OP patients without fractures (OR, 6.20; 95% CI, 4.83 to 7.96; *P*-value<0.0001). Moreover, hip fractures exhibited a higher fever risk, compared to OP patients without fractures (OR, 2.02; 95% CI, 1.37 to 2.97; *P*-value = 0.0004). Other fractures exhibited an enhanced fever risk, relative to OP patients without fractures (OR, 2.53; 95% CI, 1.84 to 3.48; *P*-value<0.0001). OP patients with hypertension experienced a lower fever risk (OR, 0.77; 95% CI, 0.63 to 0.93; *P*-value = 0.0073). Fifthly, calcitonin usage enhanced fever risk (OR, 3.33; 95% CI, 2.65 to 4.17; *P*-value<0.0001). In terms of the laboratory test covariates, the covariates that demonstrated significant positive associations with fever risk were as follows: neutrophil count, monocyte count, alanine transaminase, β-CTX, P1NP, and ferritin. Covariates that showed significant negative association with fever risk were as follows: hemoglobin, lymphocyte count, calcium, and high-density lipoprotein.

### Independent relationship between ZOL fever risk and serum 25(OH)D content

**Table 2** summarizes the independent relationship between fever risk and serum 25(OH)D levels, using multivariate linear regression analysis. We employed a two-level adjustment, based on the covariance analysis. The crude model was unadjusted, whereas, Model I was adjusted for age; gender, order of ZOL infusion, main diagnosis, season of blood collection, year of blood collection, and calcitonin usage. In contrast, Model II was adjusted for Model I plus β-CTX. Model III was adjusted for Model II plus neutrophil count, lymphocyte count, monocyte count; hemoglobin, calcium, P1NP, CCI score, and diabetes. We observed a marked negative relationship between fever risk and serum 25(OH)D levels in both Models I (OR, 0.98; 95% CI, 0.97 to 0.99; *P*-value = 0.0031) and II (OR, 0.99; 95% CI, 0.97 to 1.00; *P*-value = 0.0188). These results can be further interpreted as follows: a 10 ng/mL rise in serum 25(OH)D content was correlated with a 29% (OR, 0.71; 95% CI, 0.64 to 0.79, *P*-value = 0.0031) decrease in the odds of ZOL fever in the crude model, or a 17% (OR, 0.83; 95% CI, 0.73 to 0.94, *P*-value = 0.0031) decrease in the odds of ZOL fever in Model I, or a 14% (OR, 0.86; 95% CI, 0.76 to 0.98, *P*-value = 0.0188) decrease in the odds of ZOL fever in Model II. In Model III, the effect value was marginally insignificant (*P*-value = 0.0568) in the linear relationship, and significant (*P*-value = 0.015) in the non-linear relationship. The results are presented in **Table S3**.

**Table 2:** Association between serum 25(OH)D content and ZOL-induced fever risk in different models.

	Crude Model^a^ N=2038	Model I^b^ N=2038	Model II^c^ N=2018
		OR (95% CI) *P*-value	OR (95% CI) *P*-value	OR (95% CI) *P*-value
serum 25(OH)D level, per 1 ng/mL increase	0.97 (0.96, 0.98) <0.0001	0.98 (0.97, 0.99) 0.0031	0.99 (0.97, 1.00) 0.0188
serum 25(OH)D level, per 10 ng/mL increase	0.71 (0.64, 0.79) <0.0001	0.83 (0.73, 0.94) 0.0031	0.86 (0.76, 0.98) 0.0188

a No adjustment.

b Adjusted for age; gender, order of ZOL infusion, main diagnosis, season of blood collection, year of blood collection and calcitonin usage.

c Adjusted for Model I plus β-CTX.

OR, odds ratio; CI, confidence interval; β-CTX, beta-C-terminal telopeptide of type I collagen; 25(OH)D, 25-hydroxy vitamin D; ZOL, zoledronate.

### Threshold analysis and the spline smoothing plot

**Figure 4:**
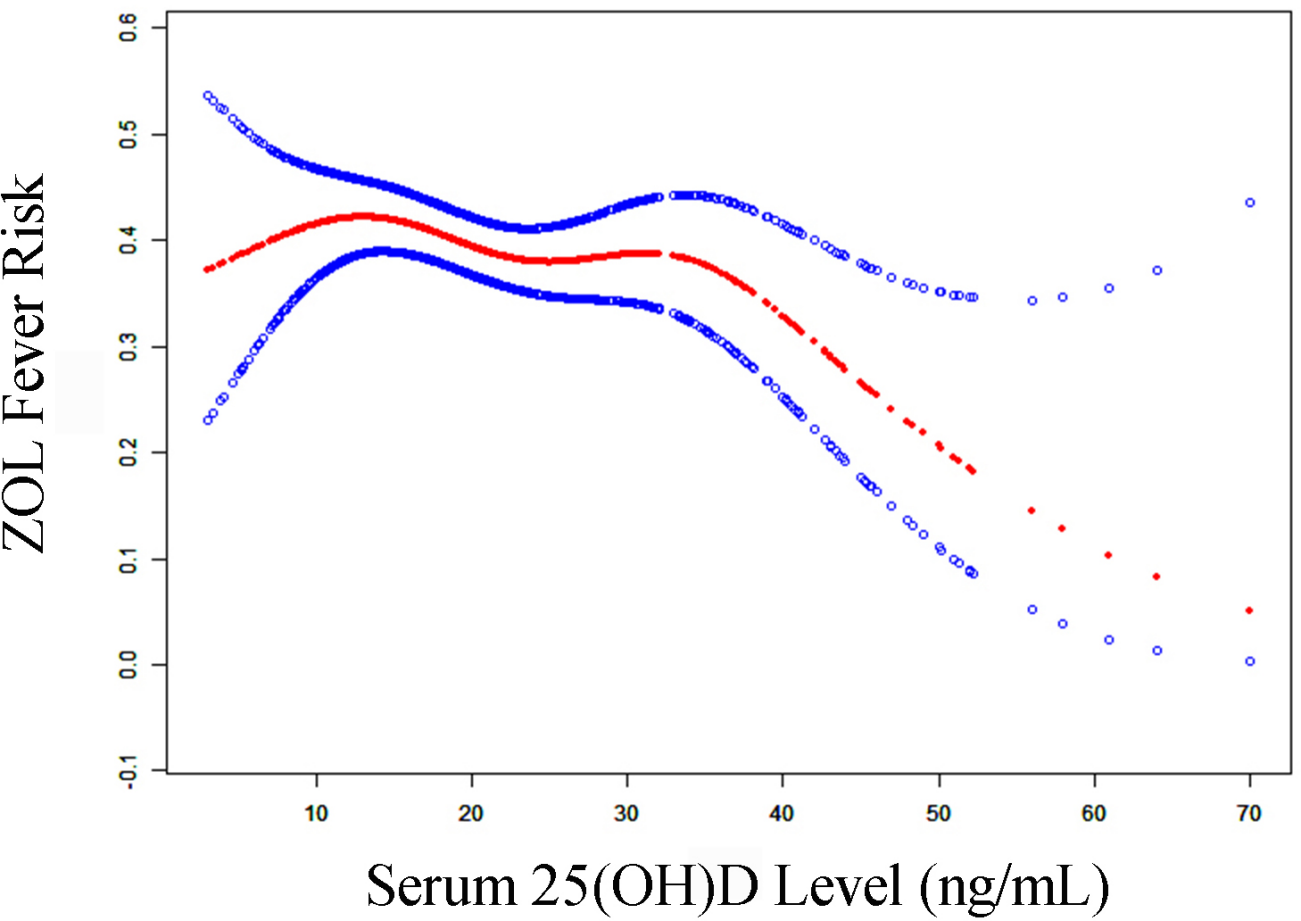
The adjusted smoothed curves of serum 25(OH)D levels and ZOL fever risk. A threshold, nonlinear correlation between serum 25(OH)D levels and ZOL fever risk, as evidenced by our generalized additive model. The red curve (middle) refers to a predicted value, and the blue curves (both sides) refer to the 95% CIs. Adjustment factors included gender, order of ZOL infusion, main diagnosis, season of blood collection, year of blood collection, calcitonin usage, and β-CTX. The turning point (K) of the curve in Model II was 35 ng/mL. CI, confidence interval; β-CTX, beta-C-terminal telopeptide of type I collagen; 25(OH)D, 25-hydroxy vitamin D; ZOL, zoledronate.

**Table 3** summarizes the threshold effect analysis that examined the correlation between serum 25(OH)D levels and ZOL fever risk in the fully adjusted Model II. The *P*-value for LRT<0.05 indicated a non-linear correlation between 25(OH)D content and ZOL fever risk. Based on the two-piecewise linear regression model, we computed the turning point (K) of the adjusted smoothed curve as 35 ng/mL of serum 25(OH)D content. Specifically, a significantly stronger negative association was observed between serum 25(OH)D content and ZOL fever risk, when the serum 25(OH)D concentration ranged from 35 to 70 ng/mL (OR, 0.93; 95% CI, 0.88 to 0.99; *P*-value = 0.0158). The adjusted spline smoothing plot visually illustrates the aforementioned results (**Figure 4**). Likewise, in the fully adjusted Model III, the result was robust (**Table S2**).

**Table 3:** Threshold effect analysis examining the correlation between serum 25(OH)D content and ZOL-induced fever risk in Model II.

	Model II^a^
		OR (95% CI) *P*-value
Model A^b^	
One line slope	0.99 (0.97, 1.00) 0.0188
Model B^c^	
Serum 25(OH)D turning point (K), ng/mL	35
<K	1.00 (0.98, 1.01) 0.5405
>K	0.93 (0.88, 0.99) 0.0158
Slope 2 – Slope 1	0.94 (0.88, 1.00) 0.0455
LRT^d^	0.03

a Adjusted for age; gender, order of ZOL infusion, main diagnosis, season of blood collection, year of blood collection, calcitonin usage and β-CTX.

b Linear analysis, P-value <0.05 indicates a linear relationship.

c Non-linear analysis.

d P-value <0.05 means Model B is significantly different from Model A, which indicates a non-linear relationship.

OR, odds ratio; CI, confidence interval; β-CTX, beta-C-terminal telopeptide of type I collagen; 25(OH)D, 25-hydroxy vitamin D; ZOL, zoledronate; LRT, logarithmic likelihood ratio test.

### Subgroup analysis

To further confirm that our findings were robust in presence of potential confounders in the fully adjusted Model II, we performed subgroup analyses while stratifying by age, gender, order of ZOL infusion, main diagnosis, season of blood collection, year of blood collection, calcitonin usage, and β-CTX. All analyses were adjusted for the aforementioned eight covariates, except for the subgroup variable. **Table 4** reveals a highly consistent pattern, and no interactions were observed based on all stratification (all *P*-values for interaction >0.05).

**Table 4:** Subgroup analyses exploring the association between serum 25(OH)D levels and ZOL-induced fever risk.

Subgroup	N	OR (95% CI) *P*-value	*P*-value for interaction
Gender			0.1426
Male	338	1.01 (0.98, 1.04) 0.4194	
Female	1757	0.98 (0.97, 0.99) 0.0054	
Age tertile			0.4336
Tertile 1 (50 - 64 y)	684	0.97 (0.94, 0.99) 0.0184	
Tertile 2 (65 - 71 y)	680	1.00 (0.97, 1.02) 0.7039	
Tertile 3 (72 - 94 y)	731	0.99 (0.97, 1.01) 0.1646	
Order of ZOL infusion			0.3334
1st	1681	0.98 (0.97, 1.00) 0.0146	
2nd	326	1.02 (0.97, 1.06) 0.4908	
3rd	79	0.00 (0.00, 0.00) <0.0001	
4th	9	Null	
Main diagnosis			0.0658
OP without fractures	1422	0.98 (0.96, 0.99) 0.0054	
OPF	673	1.00 (0.98, 1.02) 0.9888	
Season of blood collection			0.8063
Spring (March, April and May)	519	0.98 (0.95, 1.01) 0.1687	
Summer (June, July and August)	518	0.99 (0.97, 1.02) 0.5681	
Autumn (September, October and November)	612	0.99 (0.97, 1.02) 0.5019	
Winter (December, January and February)	446	0.97 (0.94, 1.00) 0.0289	
Year of blood collection			0.7124
2015	21	Null	
2016	32	Null	
2017	48	0.98 (0.90, 1.06) 0.5692	
2018	129	0.97 (0.92, 1.02) 0.1940	
2019	483	0.98 (0.95, 1.01) 0.1297	
2020	583	0.98 (0.96, 1.01) 0.1361	
2021	706	0.99 (0.97, 1.01) 0.4216	
2022	93	1.00 (0.94, 1.06) 0.9615	
Calcitonin usage			0.0626
No	1685	0.98 (0.97, 1.00) 0.0145	
Yes	410	0.99 (0.97, 1.02) 0.7178	
β-CTX tertile			0.0506
Tertile 1 (0.01 - 0.232 ng/mL)	692	0.97 (0.94, 0.99) 0.0109	
Tertile 2 (0.233 - 0.483 ng/mL)	691	0.99 (0.97, 1.01) 0.4871	
Tertile 3 (0.484 - 2.26 ng/mL)	692	0.99 (0.97, 1.01) 0.3332	

Adjusted for age; gender, order of ZOL infusion, main diagnosis, season of blood collection, year of blood collection, calcitonin usage and β-CTX except the subgroup variable.

OR, odds ratio; CI, confidence interval; β-CTX, beta-C-terminal telopeptide of type I collagen; 25(OH)D, 25-hydroxy vitamin D; ZOL, zoledronate.

### Sensitivity analysis

As part of our sensitivity analysis, we assessed APR *via* CRP measurements during 3 consecutive days post ZOL infusion, instead of body temperature. **Table S4** illustrates that the CRP^+^ (CRP>0.8mg/dL) OP patients exhibited markedly reduced 25(OH)D content, relative to the CRP^-^ (CRP<=0.8 mg/dL) OP patients (*P-*value =0.006). **Table 5** reveals the correlation between serum 25(OH)D content and CRP^+^ risk in different models. Although the sample size of the sensitivity analysis was relatively small, the results were robust, and verified the results of the main analysis.

**Table 5:** Association between serum 25(OH)D levels and CRP^+^ (CRP >0.8mg/dL) risk in various models.^a^

	Crude Model^b^ N=556	Model I^c^ N=556	Model II^d^ N=547
	OR (95% CI) *P*-value	OR (95% CI) *P*-value	OR (95% CI) *P*-value
serum 25(OH)D level, per 1 ng/mL increase	0.97 (0.95, 0.99) 0.0059	0.97 (0.95, 1.00) 0.0304	0.97 (0.95, 1.00) 0.0402
serum 25(OH)D level, per 10 ng/mL increase	0.73 (0.58, 0.91) 0.0059	0.76 (0.59, 0.97) 0.0304	0.76 (0.59, 0.99) 0.0402

a The continuous CRP data was classified as the dichotomous data as follows: ≤0.8 mg/dL represented CRP^-^, and >0.8mg/dL represented CRP^+^.

b No adjustment.

c Adjusted for age; gender, order of ZOL infusion, main diagnosis, season of blood collection, year of blood collection and calcitonin usage.

d Adjusted for Model I plus β-CTX.

OR, odds ratio; CI, confidence interval; β-CTX, beta-C-terminal telopeptide of type I collagen; 25(OH)D, 25-hydroxy vitamin D; C-reactive protein, CRP.

## Discussion

This investigation was by far the largest of its kind in China, and it demonstrated a strong independent and negative correlation between serum 25(OH)D content and ZOL fever risk. Our research was based on the full use of real-world clinical data, and the inclusion of some new covariates that may influence fever risk during analysis, namely, serum β-CTX levels and calcitonin usage. In different adjusted models, the negative linear relationship remained stable. We also observed a second non-linear correlation between 25(OH)D content and ZOL fever risk, and the turning point of the adjusted smoothed curve was 35 ng/mL for serum 25(OH)D levels. These evidences indicated that serum 25(OH)D offered a certain level of protection against ZOL-induced APR fever in the daily clinical practice setting, and it became more significant after serum 25(OH)D levels reached 35 ng/mL.

Even though our findings did not provide a strong causal association between 25(OH)D content and APR, and a randomized, controlled trial (RCT) is needed to validate our findings, a potential relationship between the two is still of marked significance. Bertoldo et al. proposed this hypothesis in an earlier paper (11). ZOL is a new generation nitrogen-based bisphosphonate (N-BP), and several reports suggested that an intravenous administration of N-BPs are taken up by monocytes and dendritic cells, which, in turn, suppresses farnesyl pyrophosphate (FPP) synthase activity, thereby, accumulating intracellular metabolites like isopentenylpyrophosphate (IPP) and dimethylallyl diphosphate (DMAPP) upstream of the FPP synthase in the mevalonate network. These metabolites are robust gamma-delta T cell receptor agonists, which activate the gamma-delta T cells, and formation of proinflammatory cytokines like interleukin-6 (IL-6), tumor necrosis factor alpha (TNF- alpha), and interferon- gamma (IFN-gamma), which are crucial for APR pathogenesis by the same lymphocytes (23–25). Vitamin D is a steroid hormone that not only contributes to the calcium/phosphate metabolism, but also negatively regulates the adaptive immune system. Moreover, it promotes innate immunity through antimicrobial peptide synthesis (26, 27). A prior report revealed that the vitamin D receptor (VDR) expression is markedly enhanced in gamma-delta T cells. Particularly, BP ligands like IPP and DMAPP markedly enhances VDR levels in the gamma-delta T cells (28). Vitamin D is a potent regulator of gamma-delta T cell response, and it selectively downregulates its inflammatory properties, and inhibits the expression of proinflammatory cytokines following IPP-based activation (28).

Our analysis also revealed that the 25(OH)D content above 35 ng/mL is likely necessary to prevent ZOL-triggered fever. At present, the data on the optimal 25(OH)D level for immunomodulation is rather scarce. Bischoff-Ferrari et al. presented a combined result of multiple studies evaluating the serum 25(OH)D threshold with regards to bone mineral density (BMD), lower-extremity function, dental health, risk of falls, fractures, and colorectal cancer (29). In terms of all analyzed endpoints, the most optimal serum 25(OH)D concentration begins at 30 ng/mL, and the best range is between 36 and 40 ng/Ml (29). This result is consistent with our study, and corroborates with the data by Bertoldo et al., which indicated that a prime amount of 25(OH) (>40 ng/mL) must be obtained prior to N-BPs administration (11). However, the average serum 25(OH)D was 21.15 ng/mL in this study, and the value was even lower in the winter and spring (19.70 ng/mL), or before the first infusion of ZOL (20.47ng/mL). These values were well below the 35 ng/mL threshold that we recommend in this paper.

Alternately, there is much controversy regarding the suitable upper limit for serum 25(OH)D. In addition, the safety of elevated serum 25(OH)D levels among various patient populations remains inconclusive. A logical upper limit, computed from the concentration of 25(OH)D in sun-exposed healthy young adults, is 50 ng/mL, until more information is obtained (15). Given these evidences, we speculated that 35-50 ng/mL of serum 25(OH)D may be the target range needed to prevent ZOL-induced fever. In terms of the amount of vitamin D supplement, studies involving older individuals revealed that 25(OH)D levels can be enhanced by 20-26 ng/mL to a mean of 40 ng/mL using only 800 IU vitamin D per day (30, 31). Multiple scientific organizations suggested that adults 50 years and older must consume a minimum of 1,000 IU of vitamin D a day (15). The National Academy of Medicine suggested that 4,000 IU of vitamin D once a day is a safe upper limit for the general population (32, 33). Hence, we suggest a daily vitamin D dosage above 1,000 IU for OP patients prior to their first ZOL infusion.

Based on prior research, fever is less prevalent after the second and third ZOL infusions. In fact, fever incidences within three days post ZOL infusion in the aforementioned study ranged from 45.73, 13.65, and 5.26% after infusions 1 to 3, respectively. These incidences were slightly higher than the incidences reported in the ZONE study (38.8% after the first infusion and 7.4% after the second infusion), which evaluated Japanese postmenopausal women with OP (34), and were higher relative to the HORIZON PFT study (20% after the first, 4% after the second, and 1% after the third infusion) (8). These discrepancies may be the result of any of the following three factors: Firstly, a difference in fever assessment methodology can produce different results. The HORIZON PFT study recorded self-reported fever, whereas, we actively recorded patient body temperature for analysis in our research. This may have contributed to the reduced fever incidence in the PFT investigation, relative to our work. Secondly, the PFT subgroup analysis was stratified by various racial groups. Those most at risk were non-Japanese Asians (including Chinese) and Pacific Islanders, and they carried a univariate OR of 2.20 and 3.39, after adjustment of other variables in the model (8). The racial differences in APR prevalence potentially reflect the differing thresholds for the reporting and recording of adverse events, or it may represent race-based alterations in cytokine formation or activity. Thirdly, although the baseline 25(OH)D levels of the PFT study were not reported, the baseline levels were about 26 ng/mL in the ZONE study, which were higher than the serum 25(OH)D levels before the first (20.47 ng/mL) and second infusions (23.47 ng/mL) in our research. The baseline 25(OH)D levels prior to ZOL infusion may influence the APR severity to ZOL in patients with OP.

Owing to a suspected analgesic influence (35, 36), we provided a short-term calcitonin administration to OP patients receiving their first ZOL infusion. However, the increased risk of ZOL fever with previous use of calcitonin in our study was surprising, which is contrary to the results reported by Reid (8). Common complications of parenterally infused calcitonin are as follows: nausea, injection site inflammatory reactions, and vasomotor complications like sweating and flushing (15). Beyond these effects, we observed no published evidences on the relationships between elevated fever risk and a combined treatment of ZOL and calcitonin. In addition, uncovering a potential mechanism is very challenging. Since a combined treatment of calcitonin and BP can rapidly and effectively reduce blood calcium levels (37), we hypothesized that the lower calcium concentration induces a transient secondary elevation in parathyroid hormone (PTH) concentration. PTH, in turn, elevates IL-6 synthesis within osteoblasts, and acts as a bone resorbing agent (38, 39). Elevated serum IL-6 concentration is prevalent in individuals with primary or secondary hyperparathyroidism (40, 41). IL-6, as mentioned above, is associated with ZOL-induced APR pathogenesis (42–45). Thus, a combined treatment of ZOL and calcitonin can likely increase the potential for side effects. Of note, before our data is confirmed, the association between fever and a combined treatment of ZOL and calcitonin require further exploration in the near future.

We did not observe any NSAID-based protection against ZOL-induced fever, despite publications reporting a strong likelihood of APR reduction following ZOL infusion with NSAID usage (37, 46, 47). However, the NSAID usage in our present investigation was not only based on prophylactic therapy, but was instead used to treat other medical conditions related to persistent pain and/or inflammation, which may further complicate patient condition. To improve the reliability of the results, future research should use the prophylactic use of NSAIDs as a covariate. The lack of relationship between statin usage and APR risk was somewhat expected due to a similar report by Reid (8). Given that the present investigation included a relatively small quantity of statin users, additional examinations are warranted to determine its relation, if any, to the regulation of APR risk.

Our conclusions can greatly impact clinical practice. Identification of APR risk factors can potentially prevent APR occurrence and enhance tolerability of intravenous ZOL. Particularly, a “vitamin D supplemental period” is essential prior to ZOL infusion, particularly the first ZOL infusion, such that an appropriate 25(OH)D (probably>35 ng/mL) level is obtained to prevent ZOL-induced APR. Patients in high altitude or during winter and spring (the Northern Hemisphere) tend to have reduced baseline vitamin D levels (48, 49), and may require an extension of this supplemental period to increase the amount of vitamin D supplementation before ZOL infusion.

According to the findings of the univariate analysis, OP patients who have higher 25(OH)D, hemoglobin, lymphocyte counts, calcium, high-density lipoprotein levels, or have lower neutrophil counts, monocyte counts, alanine transaminase, β-CTX, P1NP, and ferritin levels, or who have had previous ZOL infusions, are female, with no fractures, with hypertension, and no calcitonin use, and if the season is autumn (October to November) may have less risk of ZOL-induced fever. These variables can be selected as candidate predictors of ZOL-induced fever. We have recently published an article predicting the risk of ZOL-induced fever (50). Knowledge of these predictors allows for early intervention in patients at high risk for fever. In addition, the risk/protective factors we have identified may provide clinical evidence for the future development of new prediction models.

Our research has a number of strengths. First, certain potential confounders, such as, infection and young OP patients (age<50 years) were excluded prior to analysis. Moreover, known confounding factors, namely, age, gender, order of ZOL infusion, main diagnosis, season of blood collection, year of blood collection, calcitonin usage, and β-CTX were corrected using multiple logistic regression analyses. Second, we generated a generalized linear model to assess the linear association between serum 25(OH)D levels and ZOL-induced fever risk, and employed GAM to identify a nonlinear association between the two variables. GAM holds strong benefits in terms of nonlinear associations, and it performs adequate non-parametric smoothing and fitting of data to a regression spline. Therefore, using GAM, we were able to better identify the true association between exposure and endpoint. Third, fever was assessed using quantifiable maximum axillary temperature or CRP, rather than imprecise patient self-report.

However, this study also has certain limitations. Firstly, this study only estimated the ZOL-induced fever risk and did not include the four remaining symptoms of APR. Additionally, in-depth data on the prophylactic use of NSAIDs was not collected. Secondly, our study was an analytical retrospective investigation, and, thus, any observed associations did not represent causation. Therefore, specific intervention trials are necessary to support our recommendations. Thirdly, our research employed a single-center design, with a relatively small population size. Therefore, our findings may not be applicable to other biographical ethnic groups, or for intravenous BP other than ZOL. Given these limitations, we recommend additional investigations, involving large-scale, multi-center RCTs, and using people of different races, to better ensure robustness of our study results.

## Conclusions

In summary, this study demonstrated an independent negative correlation between serum 25(OH)D content and ZOL fever risk. According to our findings, 25(OH)D above 35 ng/mL may be more effective in preventing ZOL-induced APR. If this is confirmed, a “vitamin D supplemental period” is warranted prior to ZOL infusion, particularly the first ZOL infusion, to ensure appropriate 25(OH)D levels that protect against ZOL-induced fever.

## Data availability statement

The raw data supporting the conclusions of this article will be made available by the authors, without undue reservation.

## Ethics statement

The studies involving human participants were reviewed and approved by the Affiliated Kunshan Hospital of Jiangsu University. The ethics committee waived the requirement of written informed consent for participation.

## Author contributions

Study design: KL and QS. Study conduct: CL and KL. Data collection: Y-QG. Data analysis: KL. Data interpretation: CL and QS. Drafting manuscript: KL. Revising manuscript content: QS and KL. Approving final version of manuscript: KL, CL, QS and Y-QG. KL and CL take responsibility for the integrity of the data analysis. The corresponding author attests that all listed authors meet authorship criteria and that no others meeting the criteria have been omitted. All authors contributed to the article and approved the submitted version.

## Funding

The study was supported by National Natural Science Foundation of China (CN) (82172441), Scientific Research Project of Gusu School of Nanjing Medical University (CN) (GSKY20210244), Clinical Medical Science and Technology Development Fund of Jiangsu University (CN) (JLY2021048) and Suzhou Key Clinical Diagnosis and Treatment Technology Project (CN) (LCZX202024).

## Conflict of interest

The authors declare that the research was conducted in the absence of any commercial or financial relationships that could be construed as a potential conflict of interest.

## Publisher’s note

All claims expressed in this article are solely those of the authors and do not necessarily represent those of their affiliated organizations, or those of the publisher, the editors and the reviewers. Any product that may be evaluated in this article, or claim that may be made by its manufacturer, is not guaranteed or endorsed by the publisher.
