# Supplementary material for: Association between vitamin D and zoledronate-induced acute-phase response fever risk in osteoporotic patients
Source: Front Endocrinol (Lausanne). 2022 Oct 10;13:991913. doi: 10.3389/fendo.2022.991913 (PMC9589500; doi:10.3389/fendo.2022.991913)
Supplement: Supplementary file 1 [file Table_1.pdf]

Table S1. Temporal trends of changes in serum 25(OH)D levels in patients with osteoporosis

Variables	N	Mean ± SD (ng/mL)	β	95%CI Low	95%CI Up	*P*-value[a]
Semi-Annual						
Winter and Spring (December to May)	965	19.70 ± 8.55	Reference			
Summer and Autumn (June to November)	1130	22.39 ± 8.91	2.69	1.94	3.44	<0.0001
Season						
Spring (March, April and May)	519	19.58 ± 8.16	Reference			
Summer (June, July and August)	518	21.81 ± 8.67	2.24	1.17	3.3	<0.0001
Autumn (September, October and November)	612	22.88 ± 9.09	3.3	2.28	4.32	<0.0001
Winter (December, January and February)	446	19.84 ± 8.98	0.27	-0.84	1.38	0.6332
Month						
January	132	19.32 ± 9.35	Reference			
February	105	20.19 ± 10.86	0.87	-1.36	3.1	0.4458
March	145	17.64 ± 7.14	-1.68	-3.73	0.37	0.1089
April	176	20.61 ± 8.12	1.29	-0.67	3.25	0.1976
May	198	20.07 ± 8.68	0.75	-1.17	2.66	0.4438
June	162	21.60 ± 9.69	2.28	0.28	4.28	0.0255
July	192	20.40 ± 7.66	1.08	-0.85	3	0.2739
August	164	23.68 ± 8.44	4.36	2.37	6.35	<0.0001
September	188	23.91 ± 8.63	4.59	2.66	6.53	<0.0001
October	189	22.96 ± 8.44	3.64	1.71	5.58	0.0002
November	235	21.98 ± 9.85	2.66	0.8	4.51	0.005
December	209	20.00 ± 7.63	0.68	-1.21	2.58	0.4808

Abbreviations: 25(OH)D, 25-hydroxy vitamin D; CI, confidence interval; SD, standard deviation.

[a]Crude associations between the time of blood collection and serum 25(OH)D content.
