# Supplementary material for: Association between vitamin D and zoledronate-induced acute-phase response fever risk in osteoporotic patients
Source: Front Endocrinol (Lausanne). 2022 Oct 10;13:991913. doi: 10.3389/fendo.2022.991913 (PMC9589500; doi:10.3389/fendo.2022.991913)
Supplement: Supplementary file 2 [file Table_2.pdf]

Table S2. Association of time variation with ZOL-induced fever risk

Variables	N	%	Odds ratio	95%CI Low	95%CI Upp	*P*-value[a]
Semi-Annual						
Winter and Spring (December to May)	920	57.5	Reference			
Summer and Autumn (June to November)	1118	54.74	0.89	0.75	1.07	0.2118
Season						
Spring (March, April and May)	486	54.53	Reference			
Summer (June, July and August)	517	56.67	1.09	0.85	1.40	0.4941
Autumn (September, October and November)	601	53.08	0.94	0.74	1.20	0.6339
Winter (December, January and February)	434	60.83	1.30	1.00	1.68	0.0537
Month						
January	128	62.50	Reference			
February	99	63.64	1.05	0.61	1.81	0.8604
March	124	57.26	0.80	0.49	1.33	0.3962
April	167	56.29	0.77	0.48	1.24	0.2827
May	195	51.28	0.63	0.4	1.00	0.0477
June	161	57.14	0.80	0.50	1.29	0.357
July	192	54.69	0.72	0.46	1.14	0.1662
August	164	58.54	0.85	0.53	1.36	0.4924
September	184	61.96	0.98	0.61	1.56	0.9224
October	185	47.03	0.53	0.34	0.84	**0.0073**
November	232	50.86	0.62	0.40	0.97	**0.0342**
December	207	58.45	0.84	0.54	1.33	0.4628

Abbreviations: ZOL, zoledronate; CI, confidence interval.

[a]Crude associations between the time of blood collection and ZOL-induced fever risk.
