# Supplementary material for: Association between vitamin D and zoledronate-induced acute-phase response fever risk in osteoporotic patients
Source: Front Endocrinol (Lausanne). 2022 Oct 10;13:991913. doi: 10.3389/fendo.2022.991913 (PMC9589500; doi:10.3389/fendo.2022.991913)
Supplement: Supplementary file 3 [file Table_3.pdf]

Table S3. The relationship between serum 25(OH)D levels and ZOL-induced fever risk in Model III.[a]

	Model III[a] N=1949 OR (95% CI) *P*-value
Model A[b]	
One line slope	0.99 (0.97, 1.00) 0.0568
Model B[c]	
Serum 25(OH)D turning point (K), ng/mL	36
<K	1.00 (0.98, 1.01) 0.8595
>K	0.92 (0.86, 0.98) 0.0148
Slope 2 – Slope 1	0.92 (0.86, 0.99) 0.0293
LRT[d]	0.015

[a]Adjusted for age; gender, order of ZOL infusion, main diagnosis, season of blood collection, year of blood collection, calcitonin usage and β-CTX, neutrophil count, lymphocyte count, monocyte count; hemoglobin, calcium, P1NP, CCI score and diabetes.
[b]Linear analysis, *P*-value <0.05 indicates a linear relationship.
[c]Non-linear analysis.
[d]*P*-value <0.05 means Model B is significantly different from Model A, which indicates a non-linear relationship.

Abbreviations: OR, odds ratio; CI, confidence interval; β-CTX, beta-C-terminal telopeptide of type I collagen; P1NP, procollagen type I N-terminal propeptide; 25(OH)D, 25-hydroxy vitamin D; ZOL, zoledronate; CCI, Charlson comorbidity index; LRT, logarithmic likelihood ratio test.
