# Supplementary material for: Association between vitamin D and zoledronate-induced acute-phase response fever risk in osteoporotic patients
Source: Front Endocrinol (Lausanne). 2022 Oct 10;13:991913. doi: 10.3389/fendo.2022.991913 (PMC9589500; doi:10.3389/fendo.2022.991913)
Supplement: Supplementary file 4 [file Table_4.pdf]

Table S4. Serum 25(OH)D levels stratified by CRP

	CRP^- (CRP ≤0.8 mg/dL) (N) Mean (SD) Median (Q1-Q3)	CRP^+ (CRP >0.8mg/dL) (N) Mean (SD) Median (Q1-Q3)	Standardize diff.	*P*-value
Serum 25(OH)D level, ng/mL	(378) 21.72 (8.92) 20.32 (15.20-26.00)	(175) 19.52 (8.21) 19.00 (13.00-24.00)	0.26	0.006

No adjustment. The continuous CRP data was classified as the dichotomous data as follows: ≤0.8 mg/dL represented CRP^-, and >0.8mg/dL represented CRP^+.

Abbreviations: SD, standard deviation; Q1, first quartile; Q3, third quartile; 25(OH)D, 25-hydroxy vitamin D; C-reactive protein, CRP.
